# Sexual Differentiation and Primordial Germ Cell Distribution in the Early Horse Fetus

**DOI:** 10.3390/ani11082422

**Published:** 2021-08-17

**Authors:** Dragos Scarlet, Stephan Handschuh, Ursula Reichart, Giorgia Podico, Robyn E. Ellerbrock, Sebastián Demyda-Peyrás, Igor F. Canisso, Ingrid Walter, Christine Aurich

**Affiliations:** 1 Obstetrics, Gynecology and Andrology, Department for Small Animals and Horses, University of Veterinary Medicine Vienna, Veterinärplatz 1, 1210 Vienna, Austria; 2 Institute of Veterinary Anatomy and Clinic of Reproductive Medicine, Vetsuisse Faculty Zürich, Winterthurerstrasse 260, 8057 Zürich, Switzerland; 3 Vetcore Facility for Research, University of Veterinary Medicine Vienna, Veterinärplatz 1, 1210 Vienna, Austria; Stephan.handschuh@vetmeduni.ac.at (S.H.); ursula.reichart@vetmeduni.ac.at (U.R.); Ingrid.walter@vetmeduni.ac.at (I.W.); 4 Department of Veterinary Clinical Medicine, College of Veterinary Medicine, University of Illinois Urbana-Champaign, Urbana, IL 61802, USA; gpodico@illinois.edu (G.P.); robyn.ellerbrock@uga.edu (R.E.E.); canisso@illinois.edu (I.F.C.); 5 Department of Comparative Biosciences, College of Veterinary Medicine, University of Illinois Urbana-Champaign, Urbana, IL 61802, USA; 6 Department of Animal Production, School of Veterinary Sciences, National University of La Plata and CONICET CCT-La Plata, Calle 60 and 118 S/N, 1900 La Plata, Argentina; sdemyda@fcv.unlp.edu.ar; 7 Institute of Pathology, Department of Pathobiology, University of Veterinary Medicine Vienna, Veterinärplatz 1, 1210 Vienna, Austria; 8 Center for Artificial Insemination and Embryo Transfer, University of Veterinary Medicine Vienna, Veterinärplatz 1, 1210 Vienna, Austria; Christine.Aurich@vetmeduni.ac.at

**Keywords:** gonad, germ cell, genital duct, fetus, horse

## Abstract

**Simple Summary:**

In horses, gonadal development and sexual differentiation occur during early fetal life. This is accompanied by primordial germ cell differentiation and migration to the gonad site. However, little is known about the time when these processes take place and the mechanisms behind them. Additionally, no information is available regarding number and distribution of primordial germ cells in the equine gonad. During development and differentiation, gonads can be affected by stressors causing infertility, but this condition can only be diagnosed in post-pubertal animals. Herein we show that equine gonads develop asynchronously between male and female fetuses, and that the number and distribution of primordial germ cells is influenced by fetal sex. By day 45 of pregnancy, equine gonads were clearly differentiated, while migration of primordial germ cells still occurred at this stage. No sign of genital duct regression was seen until day 60 of pregnancy.

**Abstract:**

It was the aim of this study to characterize the development of the gonads and genital ducts in the equine fetus around the time of sexual differentiation. This included the identification and localization of the primordial germ cell population. Equine fetuses between 45 and 60 days of gestation were evaluated using a combination of micro-computed tomography scanning, immunohistochemistry, and multiplex immunofluorescence. Fetal gonads increased in size 23-fold from 45 to 60 days of gestation, and an even greater increase was observed in the metanephros volume. Signs of mesonephros atrophy were detected during this time. Tubular structures of the fetal testes were present from day 50 onwards, whereas cell clusters dominated in the fetal ovary. The genital ducts were well-differentiated and presented a lumen in all samples. No sign of mesonephric or paramesonephric duct degeneration was detected. Expression of AMH was strong in the fetal testes but absent in ovaries. Irrespective of sex, primordial germ cells selectively expressed LIN28. Migration of primordial germ cells from the mesonephros to the gonad was detected at 45 days, but not at 60 days of development. Their number and distribution within the gonad were influenced (*p* < 0.05) by fetal sex. Most primordial germ cells (86.8 ± 3.2% in females and 84.6 ± 4.7% in males) were characterized as pluripotent according to co-localization with CD117. However, only a very small percentage of primordial germ cells were proliferating (7.5 ± 1.7% in females and 3.2 ± 1.2% in males) based on co-localization with Ki67. It can be concluded that gonadal sexual differentiation in the horse occurs asynchronously with regard to sex but already before 45 days of gestation.

## 1. Introduction

Sexual differentiation of a fetus is a complex process initiated in a subset of somatic cells by the testis-determining factor, which originates from the SRY gene. Testis-determining factor then stimulates differentiation of a subset of somatic cells into Sertoli cells and their aggregation into primitive seminiferous tubules [1]. In these newly formed fetal testes, Sertoli cells secrete anti-Müllerian hormone (AMH), and Leydig cells produce testosterone. These hormones are responsible for developing the mesonephric ducts, masculinization of the external genitalia, and regression of the paramesonephric ducts [2]. Compared to the male gonad, morphological changes in fetal ovaries such as somatic cell proliferation, migration, and vascularization are less pronounced at similar fetal ages [3]. There is also an asynchrony in sexual differentiation between sexes as has been demonstrated in humans, cattle, dogs, and pigs [4,5,6,7].

There is a plethora of information regarding the role of fetal gonads in equine pregnancy [8,9,10,11]. However, there is only limited knowledge with regard to early fetal gonadal development and sexual differentiation in this species. An initial study suggested that sexual differentiation of the equine gonads occurs between 39 and 45 days of gestation [12]. In a recent study, non-sexually differentiated gonads in horse fetuses were still detected on day 40 of gestation [13]. It is worth mentioning that, in this species, the earliest time for reliable fetal sex determination by transrectal ultrasonography is at 59 days [14]. In cattle, during early gonadal development, a mesenchymal thickening occurs on the mesonephros’ ventromedial side covered by a mesothelium [15]. Intermediate filaments such as vimentin and cytokeratins are involved in tissue organization and differentiation and contribute to establishing the cytoskeleton [16]. Development-specific expression patterns of keratins and vimentin were detected in the gonads of human and bovine conceptuses [17] suggesting involvement in the control of primordial germ cell (PGC) apoptosis, early differentiation of gonadal stroma cells and cell migration [18]. In male gonads of rats and mice, AMH synthesis during early sex differentiation is associated with down-regulation of keratins and laminin [19,20], whereas in females, laminin, a major glycoprotein in basal lamina, is also involved in primordial follicle assembly [21].

The paramesonephric (Müllerian) and mesonephric (Wolffian) ducts display an asynchronous regression pattern similar to the asynchronous gonadal sexual differentiation in males and females [22]. Regardless of the chromosomal sex, the paramesonephric and mesonephric ducts are present during early fetal development. The paramesonephric duct only develops in the presence of a mesonephric duct [23]; it does, however, develop without a cellular contribution of the mesonephric duct [24]. Initially, the paramesonephric duct is mesenchymal in nature and expresses vimentin, whereas later it differentiates and expresses standard epithelial cell markers such as pan-cytokeratin [25]. It has recently been suggested that the origin of the paramesonephric duct epithelium cells is the mesonephric epithelium [25]; however, the mesonephric duct differs from the early paramesonephric duct in that it expresses pan-cytokeratin and lacks expression of vimentin [26].

As shown in other species, germ cell development and differentiation is a complex process associated with downregulation of stem cell-associated genes (e.g., *OCT4* and *KIT*) and upregulation of markers of germ cell differentiation and meiosis (e.g., *VASA*, *STRA8*, and *SYCP3*) [27,28]. In humans, PGCs were detected in fetal gonads from eight weeks of gestation and these cells selectively express LIN28 from that stage onwards until germ cells enter meiosis at 16 weeks of gestation [29,30]. The presence of LIN28 is necessary for normal development of the PGC population in vivo and for derivation of PGC-like cells from embryonic stem cells in vitro in mice [31]. Ovarian cancer cells [32], as well as a rare population of adult human spermatogonia [33], express LIN28. In horses, low numbers of putative PGCs, identified using alkaline phosphatase (AP), were detected outside the gonads but not intragonadally in embryos examined at 20 days of gestation [34]. Over the next 10 days of gestation, a massive increase in the total number of AP-positive cells occurred and the percentage of PGC found in the gonadal ridge increased from 4% at 22 days to 28% at 26 to 28 days [34]. To the best of our knowledge, this is the only report available on PGC populations in the early equine fetus. In the testes of adult stallions, LIN28 was reported to be a useful molecular marker to identify undifferentiated spermatogonial stem cells [35], whereas CD117, a protein encoded by the *KIT* gene, was expressed by differentiating spermatogonia [36]. In equine fetal gonads, however, neither LIN28, nor CD117 expression has yet been reported.

The objectives of the present work were to (i) quantify PGCs in the horse fetal gonad at days 45 to 60 of gestation, the presumptive time of gonadal sexual differentiation, and (ii) characterize the morphology of horse fetal gonads and genital ducts, as well as the developmental origin of the ducts at the time of sexual gonadal differentiation. We hypothesized that fetal gonadal differentiation and genital duct degeneration occur asynchronously in the horse as in other species, and that the number and distribution of PGCs is influenced by fetal sex. 

## 2. Materials and Methods

### 2.1. Sample Collection

Seventeen equine fetuses (5 males and 12 females) were collected at 45 days (*n* = 3, all females), 50 days (*n* = 2, one male and one female) and 60 days (*n* = 12, four males and eight females) of gestation, respectively. In all pregnancies, the day of ovulation (day 0, i.e., the day of disappearance of the preovulatory follicle) was determined by daily transrectal ultrasonography.

Fetuses collected at 45 and 50 days of gestation originated from client mares presented for pregnancy termination at the Centre for Artificial Insemination and Embryo Transfer of the University of Veterinary Medicine Vienna, Austria between April 2016 and December 2018. Ethical approval was therefore not required; however, the owner’s consent for inclusion of the material into scientific investigations was obtained for all cases. These five pregnancies from four clinically healthy multiparous mares (12 ± 1.0 years-old, 1 Haflinger, 3 Warmbloods) were interrupted either because of twin pregnancy (one mare on day 45) or due to unwanted pregnancies (one mare on day 45, two mares on day 50). These fetuses were collected transcervically using sterile lavage with warmed (38 °C) Ringer’s Lactate Solution. Flushes were performed with a modified endotracheal tube (inner diameter 20 mm, outer diameter 25 mm, length 1000 mm). Immediately after collection, the fetus and its accompanying fetal membranes were grossly examined, and then the fetal carcasses were submerged in 4% formaldehyde solution until processing.

Fetuses collected on day 60 of gestation originated from singleton pregnancies obtained by the Equine Theriogenology Service at the Veterinary Teaching Hospital of University of Illinois Urbana-Champaign, IL, USA. The study protocol was approved by the Institutional Animal Care and Use Committee of the University of Illinois Urbana-Champaign (protocol number 16129, Approval date 13 October 2016, valid for three years). Twelve pregnancies from nine clinically healthy light breed multiparous mares (13 ± 4.5 years-old, 2 Standardbreds, 3 Quarter Horses, 1 Tennessee Walking Horse, 1 Pony of the Americas, and 2 mixed breeds) were included in the study. Three of the nine mares were bred for a second gestation. All mares and the stallion enrolled in the study belonged to the research and teaching herd of the University of Illinois Urbana Champaign. Mares were examined via transrectal ultrasound, and PGF2α (dinoprost 5 mg/mare IM, Lutalyse, Zoetis, Parsippany, NJ, USA) was administered when a CL was detected to induce oestrus. As soon as a preovulatory follicle (>35 mm) was detected, the mare was inseminated with fresh extended semen (2 billion progressively motile sperm) from a 13-year-old Quarter Horse stallion housed at the same facility. Mares were examined every 24 h to detect ovulation, and pregnancy was detected at 14 days post ovulation, and ultrasounds were repeated at 28 and 45 days of gestation to ensure pregnancies were progressing normally. Mares were housed on pasture and supplemented with grass hay and trace minerals at the Veterinary Medical Research Farm University of Illinois Urbana-Champaign. The study was carried out between November 2017 and August 2018.

Fetal death was induced at 60 days of gestation either by a single intrauterine infusion of 500 µg cloprostenol (Estrumate, Intervet, Kenilworth, NJ, USA) diluted in 8 ml of saline (*n* = 6), or with intramuscular injections of 500 µg cloprostenol, q12 h until cessation of heartbeat (*n* = 6) for a parallel study [37]. For the infusion, an equine AI pipette was passed through the cervix, and the saline-cloprostenol solution was instilled caudally to the allantochorion membrane while taking care not to rupture this membrane [37]. For either method, fetal heart rate was monitored every six hours by transrectal ultrasound, and once a fetal heart rate was no longer detected, the mare’s perineum was aseptically cleaned, and the fetus and accompanying fetal membranes were recovered via manual transvaginal manipulation. Fetal death was detected in all mares within 48 h (range 18–48 h) after cloprostenol administration. Immediately after removal, the fetus and its membranes were grossly examined, and then all four fetal limbs were removed for a parallel study. The remaining fetal carcass (the “body”) was submerged in 50 mL of 4% formaldehyde solution and shipped to the University of Veterinary Medicine Vienna, Austria, for further processing.

### 2.2. Micro-Computed Tomography (microCT) Scanning

After fixation in formaldehyde, one female fetus from each stage of pregnancy was washed in distilled water. For enhancing X-ray contrast, fetuses were stained in Lugol’s solution (0.25% (*w*/*v*) elemental iodine and 0.5% (*w*/*v*) potassium iodide in distilled water). Based on different sample sizes, staining times varied from four days (45 days) to six days (50 and 60 days), respectively. After staining, samples were washed in distilled water to remove unbound iodine from tissues. For tomographic scanning, samples were mounted in 1.5% low melt agarose in either 15 mL or 50 mL Falcon tubes. For each specimen, we acquired an overview scan showing the whole fetus or its body, respectively. For fetuses from 45 and 50 days of gestation, the urogenital system’s interior tomography was also acquired, as some structures such as the genital ducts are too small at d45/d50 to be unambiguously identified in overview scans. Scans were acquired with an XRadia MicroXCT-400 (Carl Zeiss X-ray Microscopy, Jena, Deutschland) at either 80 kVp/100 µA or 90 kVp/88 µA using the 0.4 X detector assembly (scanning energy was chosen depending on the intensity of the iodine stain). Projection images were recorded with 15 s (overview scans) or 30 s (interior tomographies) exposure time and an angular increment of 0.16° between projections. For overview scans, isotropic voxel size in the reconstructed volumes varied from 12.3 µm (45 days) to 21.1 µm (60 days), depending on the fetal size. Voxel size of interior tomographies was 4.6 µm. Reconstructed image volumes were exported as DICOM sequences.

Image volumes were imported into Amira 6.4 (FEI SAS, part of Thermo Scientific, Waltham, MA, USA). Interior tomographies were registered to overview scans. Subsequently, the urogenital region (mesonephros, metanephros (kidneys), gonads, adrenal glands, mesonephric ducts/paramesonephric ducts, and ureters) was divided using manual digital segmentation tools such as brush and lasso. Surface models were created from segmentation masks. Overview scans and models of the urogenital organs were visualized by combining volume and surface rendering [38].

After microCT imaging, fetuses were washed in 50% ethanol (room temperature, horizontal shaking) to remove the iodine stain from tissues. The washing solution was replaced several times during this procedure until the embryo gradually discolored from brownish (iodine stain) to its original color after fixation. Once the fetuses were discolored, they were transferred to 70% ethanol. Subsequently, they were embedded in paraffin using standard dehydration and embedding protocols.

### 2.3. Immunohistochemistry

Immunolocalization of AMH, LIN28, Ki76, CD117, vimentin, laminin, and pan-cytokeratin in the fetal gonads was carried out by using primary antibodies raised against the specific proteins (Table 1). After fixation in 4% formaldehyde solution, fetuses were cut in sequential transverse slabs followed by dehydration in ethanol and embedding in paraffin. Tissues embedded in paraffin blocks were sliced into 2 µm sections, mounted on slides, and dried overnight at 37 °C. Gonads from all specimens were identified and stained with H&E for histological evaluation. For immunohistochemical staining, paraffin sections were dewaxed in xylene for 8 min and then rehydrated in descending concentrations of ethanol (100%, 96% (*v*/*v*) and 70% (*v*/*v*) at room temperature, each for 3 min. Endogenous peroxidase activity was suppressed by incubation of slides in 0.6% (*v*/*v*) H_2_O_2_ in methanol for 15 min at room temperature, followed by ten washings with tap water. Antigen retrieval was performed by heating tissue sections for 30 min in a steamer (Morphy Richards, Swinton, England) either in citrate buffer (0.01 M pH 6.0) for LIN28, Ki67, and vimentin, or in Tris-EDTA (pH 9.0) for AMH, CD117, and pan-cytokeratin, respectively. For laminin, antigen retrieval was performed by digesting the tissue with 1 mg/mL protease in PBS (Sigma Aldrich, St. Louis, MO, USA) for 20 min at room temperature, followed by two washing steps in distilled water. Nonspecific binding was blocked by incubation with 1.5% (*v*/*v*) normal goat (for LIN28, Ki67, CD117, vimentin, and pan-cytokeratin) or rabbit (for AMH) serum (Sigma Aldrich) for 30 min at room temperature. Thereafter, sections were incubated with the respective primary antibodies at 4 °C overnight in a humidified chamber. Then, sections were rinsed with PBS (pH 7.4) and incubated with secondary antibody (BrightVison Poly-HRP; ImmunoLogic Technologies, Duiven, The Netherlands) for 30 min at room temperature. Immunostaining was visualized using diaminobenzidine chromogen (Thermo Fisher). Primary antibody against AMH and CD117 was successfully used in previous studies with equine tissues [36,39]; the fetal gonad served as an internal control for LIN28, as this protein was strongly expressed and solely localized in PGCs; equine colon served as a positive control for Ki67; equine tissue microarrays were used as internal controls for laminin (basal lamina), vimentin (mesenchyme–endothelium), pan-cytokeratin (epithelium–cytoskeleton) and ß-catenin (cell–cell adhesion). Staining specificity was demonstrated by omitting the respective first antibody. All slides were evaluated by light microscopy with an Axio Imager Z2 (Carl Zeiss) at increasing magnifications from 50× to 400×, and digital images were captured using Zen 2012 (blue edition) software (Carl Zeiss).

### 2.4. Multiplex Immunofluorescence

All fetuses used for immunohistochemistry analysis were also submitted to immunofluorescence staining for assessment of proliferation and distribution of PGCs in the fetal gonads. The slides were dewaxed in xylene and rehydrated using a decreasing series of ethanol (100%, 96% (*v*/*v*) and 70% (*v*/*v*) at room temperature, each for 3 min). Endogenous peroxidases were quenched using 3% hydrogen peroxide (Merck, Darmstadt, Germany) for 60 min at room temperature, followed by washing in deionized water. Antigen retrieval was performed by heating the slides in Tris-EDTA (pH 9.0) for 30 min in a steamer. Unspecific binding sites were blocked using 10% normal goat serum (Sigma Aldrich) for 60 min at room temperature. The first primary antibody rabbit-anti-LIN28 (Table 1) was incubated overnight at 4 °C. After washing in PBS, slides were incubated with secondary antibody BrightVision-Poly-HRP anti-rabbit (Immunologic) for 60 min at room temperature, followed by incubation with Tyramide working solution 647 (Invitrogen, Carlsbad, CA, USA). Slides were then heated in 0.01 M citrate buffer for 15 min in a microwave oven to remove the primary–secondary antibody complex. Blocking of endogenous peroxidases and blocking of unspecific binding sites was repeated, and the slides were incubated with the second primary antibody rabbit-anti-CD117 (Table 1) overnight at 4 °C. The following day slides were again incubated with BrightVision-Poly-HRP anti-rabbit for 60 min at room temperature, followed by incubation with Tyramide working solution 488 (Invitrogen). Slides were then heated again in 0.01 M citrate buffer for 15 min in a microwave oven to remove the antibody complex. After blocking of endogenous peroxidases and unspecific binding sites, slides were again incubated with the third primary antibody mouse-anti-Ki67 (Table 1) overnight at 4 °C. Slides were incubated with BrightVision-Poly-HRP anti-mouse secondary antibody followed by incubation with Tyramide working solution 567 (Invitrogen). Nuclei were stained using DAPI (Sigma Aldrich) for 3 min at room temperature, washed in PBS, and mounted using Mowiol (Polysciences, Warrington, PA, USA) mounting medium and coverslip. For further analysis, slides were scanned using Pannoramic scan II (3DHISTECH, Budapest, Hungary) and gonads were identified and selected with the software Pannoramic Viewer (3DHISTECH).

The analysis of particles positive for LIN28, CD117, and Ki67, respectively, was done using the software ImageJ as reported [40]. Particles were segmented by thresholding, and particle counts and positive areas were calculated in relation to the entire area of gonad tissue. Subsequently, particles positive for LIN28, CD117 or Ki67 were analyzed with regard to overlapping regions among each other. Two proteins were considered to be co-localized when at least 50% of the area of the positive particle overlapped. Consistent image analysis was supported by using a macro covering the whole ImageJ workflow.

### 2.5. Molecular Analyses

Sex of the fetuses was confirmed using a previously described multiplex polymerase chain reaction, which also enables detection of the most important sex chromosomal abnormalities reported in horses (chimerism, Turner’s syndrome, and sex reversal syndromes) [41].

From the paraffin-embedded tissue, 3 to 9 10 µm sections (depending on the size of the cross-section) were used for DNA extraction with the QIAamp DNA FFPE Tissue Kit (Qiagen, Hilden, Germany) according to manufacturer’s protocol. DNA concentrations were measured on a DeNovix DS-11 spectrophotometer (DeNovix, Wilmington, DE, USA). Seven STR (single-tandem-repeat)-markers were amplified in a multiplex PCR using HEX and FAM labeled primers. Five markers were located on the ECAX (*LEX003*, *UCDEQ502*, *TKY38*, *LEX026*, and *TKY270*) and two markers were located on the ECAY (*EcaYH12* and *SRY*). Amplification was carried out in a 20 µL reaction including 20–60 ng of genomic DNA, 1.5–7.5 pmol of each primer pair, 0.33 mmol/L dNTPs, 2.5 mmol/L of MgCl2, 1.5 µL of 10 × PCR reaction buffer, and 1.5 U of HorsePower Taq DNA polymerase (Canvax Biotech, Cordoba, Spain). The thermal protocol included an initial denaturation at 95 °C for 10 min, 33 cycles at 94 °C for 30 s, 57 °C for 1 min, and 72 °C for 30 s, followed by 72 °C for 10 min. PCR products were genotyped by capillary electrophoresis using an Applied Biosystems 3130 xl DNA sequencer (Applied Biosystems, Foster City, CA, USA). Allele sizes were determined with the GENEMAPPER 4.0 package using a LIZ 500 bp internal size standard (Applied Biosystems).

### 2.6. Statistical Analysis

For statistical analyses, the computer software IBM SPSS statistics version 24 (IBM-SPSS, Armonck, NY, USA) was used. Data were tested for normal distribution by the Kolmogorov–Smirnov test. All data were normally distributed, therefore parametric tests were used throughout. For the analysis of LIN28, CD117, and Ki67, positive areas in the gonads and their co-localization, univariate analysis with sex as a covariate was used. Due to the limited number of samples from day 45 and day 50, only day 60 pregnancies were included. Pearson’s coefficient of correlation testing was employed to analyze the associations between markers for the triple immunofluorescence staining. Descriptive data are shown as the mean ± SEM. A *p* value < 0.05 was considered statistically significant.

## 3. Results

### 3.1. Micro-Computed Tomography 

**Figure 1:**
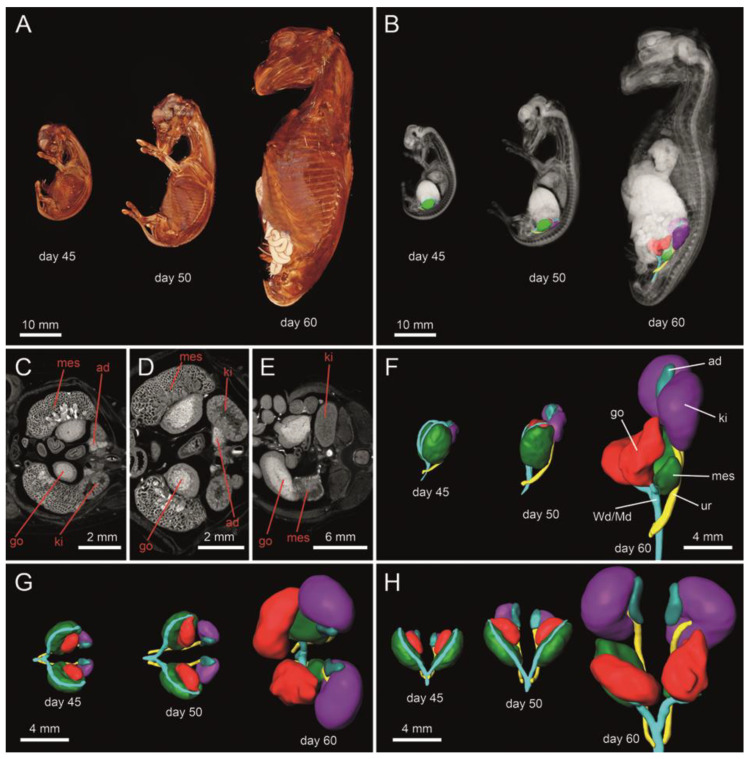
The 3D reconstruction of female equine fetuses at 45, 50 and 60 days of gestation (**A**). Structural representation of the urogenital tract in transverse section (**C**–**E**). 3D architecture of the urogenital tract (**B**,**F**–**H**) in situ (**B**), as well as from lateral (**F**), cranial (**G**) and ventral view (**H**). A well identifiable delimitation between the cortical and the medullar region can be observed in the developing gonads (**C**–**E**). Color-coded abbreviations: go, gonad (red); Wd/Md, Wolffian (mesonephric) duct/Müllerian (paramesonephric) duct (turquois green); ad, adrenal gland (blue); ki, kidney/metanephros (violet); mes, mesonephros (green); ur, ureter (yellow).

**Figure 2:**
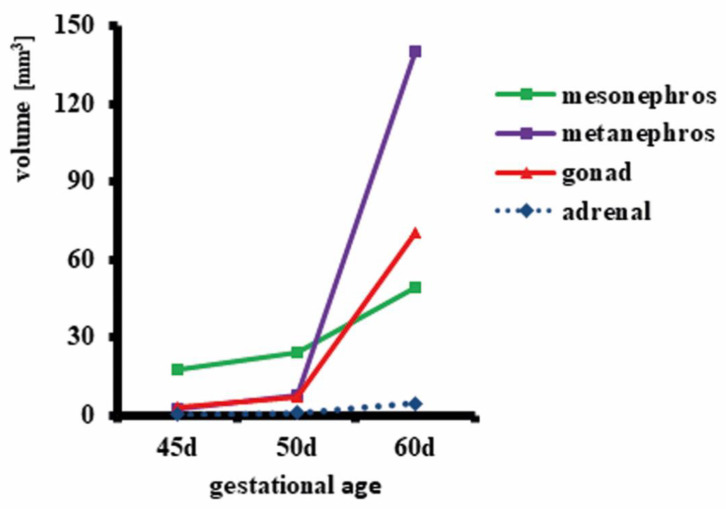
Size (volume) of mesonephros, metanephros, gonads and adrenal glands in female horse fetuses at 45, 50 and 60 days of gestation. After microCT scanning of one female fetus from each stage, image volumes were imported into Amira 6.4 and the urogenital region (mesonephros, metanephros, gonads, and adrenal glands) was divided using manual digital segmentation tools. Surface models were created from segmentation masks and volumes calculated by the software.

In all assessed fetuses, gonads were located in the sublumbar region and were connected to the mesonephros (Figure 1). The size of the gonads and metanephros increased dramatically from 45 to 60 days of gestation, yielding roughly a 60-fold volume increase for the kidneys and a 23-fold volume increase for the gonads in female fetuses (Figure 2). This could not be determined in the male sex due to the lack of male fetuses collected at 45 days. In comparison to the gonads, the mesonephros increased much less in volume between day 45 and day 60 (Figure 2). In female fetuses, microCT analysis revealed a well identifiable delimitation between the cortical and the medullar region in the developing gonads (Figure 1C–E). There were noticeable structural changes in the mesonephros, the temporary kidney organized in glomeruli and mesonephric tubules: these were present on days 45 and 50 of gestation (Figure 1C,D), but were not detectable on day 60, indicating that the mesonephros already started to undergo atrophy (Figure 1E). In addition, at 45 and 50 days of gestation, paramesonephric and mesonephric ducts extended in an anteroposterior direction, originating from the most anterior portion of the mesonephros and then running closely adjacent to the mesonephros, finally intersecting with the ureters in the caudal part of the abdomen before entering the pelvic area (Figure 1F–H). At 60 days of gestation, mesonephros retracted dorsally from the gonads and it was barely in contact with the ducts (Figure 1F).

### 3.2. Descriptive Histological Evaluations 

**Figure 3:**
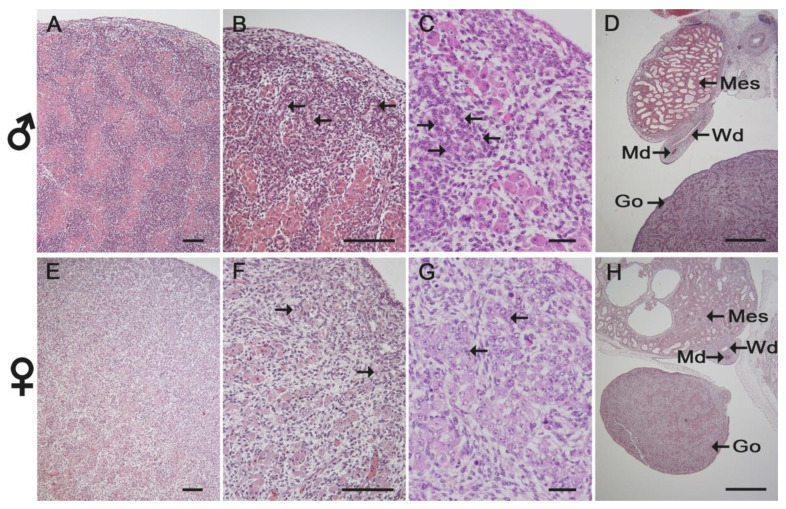
Histology (H&E staining) of male (**A**–**D**) and female (**E**–**H**) gonads and genital ducts in horse fetuses at 60 days of gestation. Seminiferous tubules (arrows in **B**,**C**) were already developed in males and evenly distributed within the gonad (**A**–**C**). The female gonad presented cord-like clusters of large epithelioid cells (arrows in **F**,**G**) mainly at its periphery (**E**–**G**). Abbreviations: Go, gonad; Wd/Md, Wolffian (mesonephric) duct/Müllerian (paramesonephric) duct; Mes, mesonephros. Scale bars in (**A**,**B**,**E**,**F**): 100 µm; (**C**,**G**): 50 µm; (**D**,**H**): 500 µm.

A single layer of flat to cuboidal cells covered all examined fetal gonads. At 50 and 60 days, tubular structures—the future seminiferous tubules—were already fully developed in males (Figure 3A–C). A high number of large interstitial cells predominated the gonadal stroma in the male fetuses. In the developing female gonad, however, large epithelioid cells were organized in cord-like clusters surrounded by connective tissue (Figure 3E–G). Irrespective of time and sex, Wolffian (mesonephric) and Müllerian (paramesonephric) ducts were clearly developed and lined by a cuboidal to columnar epithelium (Figure 3D,H). The lumen of the mesonephric ducts was consistently larger than the lumen of paramesonephric ducts.

### 3.3. Immunohistochemistry

**Figure 4:**
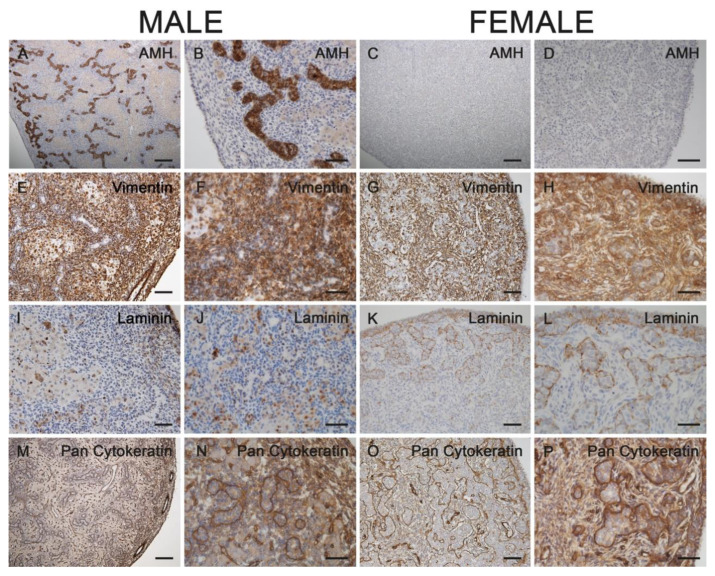
Immunostaining for AMH (**A**–**D**), vimentin (**E**–**H**), pan-cytokeratin (**I**–**L**) and laminin (**M**–**P**) in male (**A**,**B**,**E**,**F**,**I**, **J**,**M**,**N**) and female (**C**,**D**,**G**,**H**,**K**,**L**,**O**,**P**) gonads from 60-day-old horse fetuses. AMH was only expressed in the male fetal testes (**A**,**B**). Vimentin was expressed in interstitial cells of gonads from both sexes (**E**–**H**). The surface epithelium of fetal male and female gonads, as well as the cord-like structures in females, stained positive for cytokeratins (**I**–**L**). A basal lamina-like structure surrounded the developing seminiferous tubules in males and the cord-like structures in females (**M**–**P**). (**B**,**D**,**F**,**H**,**J**,**L**,**N**,**P**) represent higher magnifications of their left images. Scale bars for all pictures: 50 µm.

Expectedly, AMH was strongly expressed in cells of the future seminiferous tubules in the fetal testes but was completely absent from fetal ovaries (Figure 4A–D). The mesenchymal marker vimentin was strongly expressed in interstitial cells of gonads within the tubular or cord-like structures irrespective of fetal sex and age (Figure 4E–H). Cytokeratins, as epithelial cytoskeletal markers, were present in the surface epithelium of fetal male and female gonads at all stages as demonstrated by immunostaining with a pan-cytokeratin antibody (Figure 4I–L). In the female gonad, cord-like structures in the cortical area were stained as well. The latter signal was restricted to the basal region of cord cells. Laminin immunostaining demonstrated that a basal lamina-like structure was already surrounding the developing seminiferous tubules in males and the cord-like structures in females (Figure 4M–P). This immunostaining pattern was identical in all analyzed gonads.

**Figure 5:**
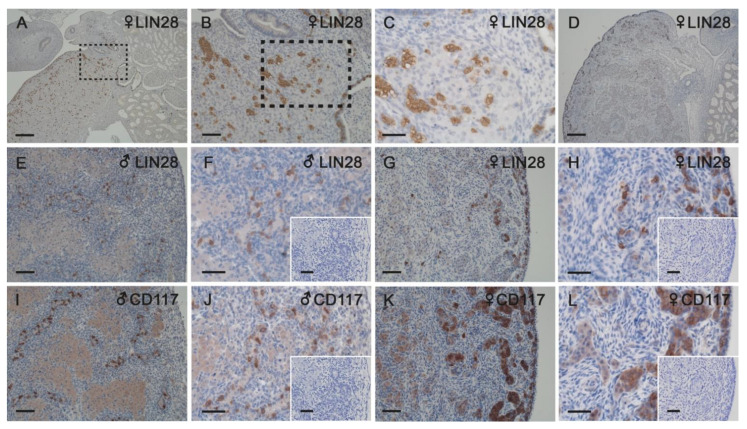
Immunostaining for LIN28 (marker for PGCs, **A**–**H**) and CD117 (marker for pluripotent cells, **I**–**L**) in female (**A**–**D**,**G**,**H**,**K**,**L**) and male (**E**,**F**,**I**,**J**) gonads from 45-day-old horse fetus (**A**–**C**) and 60-day-old horse fetuses (**D**–**L**). (**B**) Magnified image of area identified in (**A**). (**C**) Magnified image of area identified in (**B**). Inserts in **F**, **H**, **J** and **L** represent negative controls. At 45 days of development, PGCs were presumably migrating from the mesonephros to the gonad, but this was not the case at 60 days. PGCs were restricted to the tubular structures in males (**E**,**F**) and organized in cord-like structures localized in the cortical region in females (**G**,**H**). CD117 showed a similar distribution pattern to LIN28 (**I**–**L**). Scale bars in (**A**) and (**D**): 200 µm; all other pictures: 50 µm.

At 45 days of gestation, LIN28 positive cells, representing PGCs, were found scattered across the fetal gonad, but also towards the renal corpuscules of the mesonephros adjacent to the gonad (Figure 5A–C). A presumptive migration of PGCs from the mesonephros to the gonad was observed at 45 days of development, but not at 60 days (Figure 5D). In 60-day-old male gonads, PGCs were restricted to the already developed tubular structures (Figure 5E,F). In the female fetal gonad, PGCs were organized in cord-like structures localized in the cortical region (Figure 5G,H). Immunohistochemistry for the stem cell factor receptor CD117 resulted in a similar distribution pattern (Figure 5I–L). Proliferation marker Ki67 was localized in tubule-like structures and cords and in the gonadal stroma (not shown).

**Figure 6:**
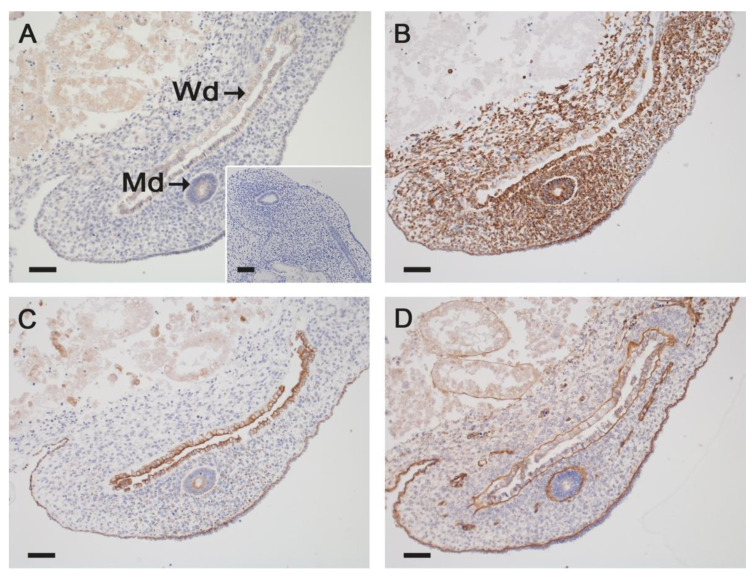
Immunostaining for ß-catenin (**A**), pan-cytokeratin (**B**), vimentin (**C**) and laminin (**D**) in Wolffian (Wd) and Müllerian (Md) ducts. Representative images from a 60-day-old female horse fetus are presented. Both ducts were positive for ß-catenin and vimentin irrespective of fetal sex (**A**,**B**). While the mesonephric ducts consistently stained positive for cytokeratin in all samples, in the paramesonephric ducts the staining ranged from only apical cytoplasmic staining to an entirely positive cytoplasm (**C**). Both ducts presented a well-developed basal lamina in both sexes (**D**). Scale bar for all pictures: 50 µm.

The epithelium of both ducts was positive for ß-catenin and vimentin in both sexes (Figure 6A,B). Different pan-cytokeratin immunostaining (Figure 6C) pattern was observed between mesonephric and paramesonephric ducts: the epithelium of mesonephric ducts was immunostained for cytokeratin in all samples, whereas the epithelium of the paramesonephric ducts ranged from only apical cytoplasmic staining to an entirely positive cytoplasm. Despite the mesenchymal origin of the paramesonephric ducts, the adjacent stroma was positive for pan-cytokeratin. A laminin-positive basal lamina-like structure was clearly developed around the mesonephric and paramesonephric ducts in both sexes at all stages (Figure 6D).

### 3.4. Immunofluorescence 

**Figure 7:**
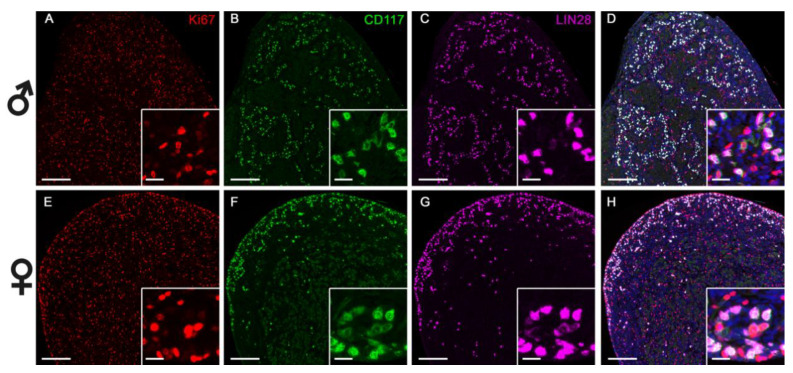
Triple immunofluorescence staining of fetal male (**A**–**D**) and female (**E**–**H**) horse gonads at 60 days of gestation. Proliferation marker Ki67 (red) is present throughout the gonad in both sexes and has a different localization than LIN28 and CD117. A distinct staining pattern depending on sex is observed for CD117 (green) and LIN28 (pink): in male gonads, CD117 and LIN28 are scattered throughout the gonad, while in females they are mainly localized at the periphery of the gonad. Scale bar: 100 µm for large pictures, 25 µm for the inserts. Blue staining denotes cell nuclei.

Primordial germinal cells in female and male gonads were LIN28+/CD117+ and localized mainly in the subcortical area in females and in the future seminiferous tubules distributed throughout the gonad in males (Figure 7). According to co-localization analysis, 86.8 ± 3.2% of LIN28+ cells in females and 84.6 ± 4.7% in males were also CD117+ (Table 2). Less LIN28+ cells (3.0 ± 0.4% vs. 4.5 ± 0.3%, *p* < 0.05) were present in female than in male 60-day-old gonads (Table 2). Of the PGCs, only a very small percentage was proliferating (3.2 ± 1.2% in males and 7.5 ± 1.7% in females, Table 2), based on co-localization with Ki67 (Figure 7). Protein expression of LIN28 and Ki67 was strongly positively correlated (r = 0.92, *p* = 0.003).

### 3.5. Molecular Analyses

The sex of all fetuses included in this study was successfully confirmed by PCR. Moreover, all analyzed fetuses were free of any of the most common chromosomal abnormalities in horses such as chimerism, Turner’s syndrome, and sex reversal syndromes (results not shown).

## 4. Discussion

This study aimed to investigate gonad sexual differentiation and genital duct development in horse fetuses from 45 to 60 days of pregnancy. During this interval, microscopic and macroscopic features of the two sexes become obvious and, by the end of it, fetal sex determination by transrectal ultrasonography examination is possible (reviewed by [14]). To the best of our knowledge, this is the first study to carry out a thorough evaluation of equine fetal gonads between 45 and 60 days of pregnancy. Herein, we employed a combination of advanced imaging and comprehensive immunostaining and fluorescence techniques to characterize this important reproductive event in the horse’s biology.

The pronounced increase in fetal gonad size, paralleled by an even greater rise in the volume of metanephros and by mesonephros atrophy, suggests that gonadal hyperplasia already starts at 45 days of gestation. An influx of interstitial cells to the horse fetal gonads after 40 days of gestation has been previously described [13], and these cells are presumably derived from the degenerating mesonephros [42]. In the male mouse, mesonephric cell migration is essential for male-specific gene expression and differentiation of Sertoli cells [43]. Tubular structures of the fetal testes were present from day 50 onwards, whereas the female fetal gonad still showed clusters of cuboidal cells at the periphery. Gonads were clearly distinct at all stages and in all fetuses assessed; these findings are consistent with a previous report involving bovine embryos at the same gestational age [4]. Primordial germ cells assembled in groups in mesenchymal compartments and were already encapsulated by a basal lamina of laminin. The genital ducts in equine fetuses herein were also well-differentiated and presented a lumen in all samples. Degeneration of the paramesonephric duct starts at the 4.5 cm stage in the cow fetus [44] and by day 40 of gestation in the male pig fetus [22]. No sign of mesonephric or paramesonephric duct degeneration was detected in the equine fetuses analyzed in our study although they were tracked from the gonad to the urogenital sinus.

Vimentin is a specific marker for mesenchymal cells; keratins are mostly found in epithelial cells, whereas laminin, a marker for basal lamina, plays an important role in the differentiation and growth of the genital ducts in many mammalian species [23,45]. In the present investigation, both ducts exhibited positive immunostaining for vimentin and showed a laminin-positive basal lamina-like structure irrespective of sex, which confirms their mesenchymal origin. We were also able to demonstrate a positive signal for pan-cytokeratin in the cranial portion of the paramesonephric ducts, suggesting an epithelial component, which is in agreement with findings in the bovine fetus [23]. An immunostaining pattern for pan-cytokeratin was detected in the mesonephric ducts in all investigated stages. Although β-catenin mediates AMH signaling for paramesonephric ducts’ regression during male sexual differentiation in mice [46], herein we could not observe any difference in the expression of this protein in the paramesonephric and mesonephric ducts. Its involvement in paramesonephric duct regression in the horse, however, cannot be totally excluded and further studies are warranted to clarify the potential role of ß-catenin in AMH signaling during sexual gonadal differentiation and sexual development in this species.

At 45 days of development, migration of PGCs from the renal corpuscules of the mesonephros to the gonad could still be observed, as described elsewhere from 20 to 30 days of gestation [34]. Recently, interstitial cells have been reported to start migrating to gonads after 40 days of gestation in equine fetuses [13]. However, presumably migrating cells in our study were LIN28+, therefore suggesting that cells in the former study were not interstitial cells. In the human fetal ovary, primordial and pre-meiotic germ cells selectively express LIN28 [28]. In horses, LIN28 mRNA expression in embryonic cells has been associated with pluripotency [47]. In the present study, our findings concur with the human literature [29] as PGCs in the fetal gonads from 45 to 60 days of gestation selectively expressed LIN28. At 60 days of gestation, PGCs organized in cord-like structures close to the fetal ovary surface. Simultaneously, the male fetal gonad presented well-delimited tubular structures and more PGCs than in females, which contrasts with findings in human gonads at a similar gestational age [6]. We detected extensive overlap between expression of LIN28 and CD117 at 60 days of gestation in both sexes. Of interest, CD117, a protein encoded by the *KIT* gene, is a surface marker for pluripotent cells which has been identified in gonocytes and preoogonia of human fetal ovaries between eight and thirteen weeks post fertilization [5]. The detection of LIN28+/CD117+ cells in the present study suggests that PGCs detected in the horse gonad at 60 days of gestation are still pluripotent and have not started meiosis yet. Further analyses using other proteins specifically expressed by pluripotent cells, (i.e., NANOG and OCT4) should confirm these results and better characterize PGCs in the horse fetal gonad.

## 5. Conclusions

To the best of our knowledge, this is the first study to investigate the morphology of fetal gonads and genital duct origin and development in the horse around the time of sexual differentiation. Results suggest that gonadal sexual differentiation in the horse occurs asynchronously between sexes and before 45 days of gestation. Furthermore, we described the primordial germ cell population and localization in gonads of both sexes and demonstrated LIN28 to be a specific marker for PGCs in the horse gonad. The temporal distribution of PGCs in the developing horse gonad and the role of LIN28 in the maintenance of the germline stem cell state needs to be investigated, as does the exact stage where the equine gonad differentiates and when ducts start degenerating.

## Figures and Tables

**Table 1:** List of antibodies used for immunohistochemistry and immunofluorescence.

Antibody	Clone	Host	Raised against (Species)	Manufacturer	Catalog #	Dilution	Target
AMH	polyclonal	Goat	human	Santa Cruz, Dallas, TX, USA	Sc-6886	1:200	Sertoli cells
LIN28	polyclonal	Rabbit	human	Abcam, Cambridge, UK	Ab46020	1:15.000	PGCs
Ki67	8D5	Mouse	human	Cell Signaling, Danvers, MA, USA	9449	1:400	Proliferating cells
CD117	polyclonal	Rabbit	human	Dako, Santa Clara, CA, USA	A4502	1:600	Pluripotent cells
Vimentin	V9	Mouse	pig	Dako	M0725	1:500	Mesenchyme–endothelium
Laminin	polyclonal	Rabbit	rat	Dako	Z0097	1:10.000	Basal lamina
Pan Cytokeratin	AE1 + AE3	Mouse	human	Cell Marque, Rocklin, CA, USA	313M-16	1:500	Epithelium–cytoskeleton
ß-catenin	9G2	Mouse	human	Acris, Herford, Deutschland	AM00020PU-N	1:400	Cell–cell adhesion
	**Tyramide Triple Immunofluorescence**						
LIN28	polyclonal	Rabbit	human	Abcam	Ab46020	1:1000	PGCs
CD117	polyclonal	Rabbit	human	Dako	A4502	1:100	Pluripotent cells
Ki67	8D5	Mouse	human	Cell Signaling	9449	1:200	Proliferating cells

**Table 2:** Percentiles of immunofluorescent staining in gonads of horse fetuses at 60 days of gestation for single markers (Ki67, CD117, and LIN28) and respective co-localization of two markers.

	Ki67	CD117	LIN28	Ki67 of LIN28	CD117 of LIN28
Male (*n* = 4)	6.2 ± 1.2	5.9 ± 1.0	4.5 ± 0.3 ^a^	3.2 ± 1.2	84.6 ± 4.7
Female (*n* = 8)	5.6 ± 0.9	3.6 ± 0.9	3.0 ± 0.4 ^b^	7.5 ± 1.7	86.8 ± 3.2

Different superscripts denote differences between sexes (*p* < 0.05).

## Data Availability

Not applicable.
